# The Binding Properties and Physiological Functions of Recoverin

**DOI:** 10.3389/fnmol.2018.00473

**Published:** 2018-12-20

**Authors:** Jingjing Zang, Stephan C. F. Neuhauss

**Affiliations:** Institute of Molecular Life Sciences, University of Zurich, Zurich, Switzerland

**Keywords:** recoverin, phototransduction cascade, G protein-coupled receptor kinase, visual pigment phosphorylation, Ca^2+^ myristoyl switch

## Abstract

Recoverin (Rcv) is a low molecular-weight, neuronal calcium sensor (NCS) primarily located in photoreceptor outer segments of the vertebrate retina. Calcium ions (Ca^2+^)-bound Rcv has been proposed to inhibit G-protein-coupled receptor kinase (GRKs) in darkness. During the light response, the Ca^2+^-free Rcv releases GRK, which in turn phosphorylates visual pigment, ultimately leading to the cessation of the visual transduction cascade. Technological advances over the last decade have contributed significantly to a deeper understanding of Rcv function. These include both biophysical and biochemical approaches that will be discussed in this review article. Furthermore, electrophysiological experiments uncovered additional functions of Rcv, such as regulation of the lifetime of Phosphodiesterase-Transducin complex. Recently, attention has been drawn to different roles in rod and cone photoreceptors.This review article focuses on Rcv binding properties to Ca^2+^, disc membrane and GRK, and its physiological functions in phototransduction and signal transmission.

## Introduction

Calcium ions (Ca^2+^) are multifaceted second messenger that regulate a large variety of signaling pathways in all cell types, including those of the brain and the retina. Specialized Ca^2+^-binding proteins, belonging to the neuronal calcium sensor (NCS) family, detect neuronal Ca^2+^ (Burgoyne et al., 2004; Weiss et al., 2010). One family member, the approximately 23-kDa protein Recoverin (Rcv), is mainly expressed in retinal photoreceptors. Like other family members, it contains four EF hand motifs and a myristoyl chain on the N-terminus. Only two of the EF hands (EF2 and EF3) bind Ca^2+^ across species (Senin et al., 2002; Lamb and Hunt, 2018). Rcv undergoes a conformational change upon Ca^2+^ binding in darkness and subsequently attaches to the disc membrane in photoreceptor outer segment (Tanaka et al., 1995; Ames et al., 1997). This in turn allows binding of G protein-coupled receptor kinases (GRK). Ca^2+^-bound Rcv inhibits GRK and thereby prevents visual pigment phosphorylation. Under bright light condition, Ca^2+^ dissociates from Rcv and the protein translocates to the inner segment. The released GRK is now able to phosphorylate and eventually deactivate the light activated visual pigment (R*; Strissel et al., 2005). This mechanism was postulated to be part of the negative Ca^2+^ feedback during light adaption. Recent studies demonstrate its additional roles in regulating PDE* lifetime and synaptic transmission (Sampath et al., 2005; Chen et al., 2012, 2015).

Here, we review recent insights into the mechanism of Rcv binding to Ca^2+^, phospholipid membranes and GRK, before focusing on the physiological importance of Rcv in regulating the phototransduction cascade and synaptic transmission.

## Ca^2+^ Binding Induced Conformational Transition

**Figure 1:**
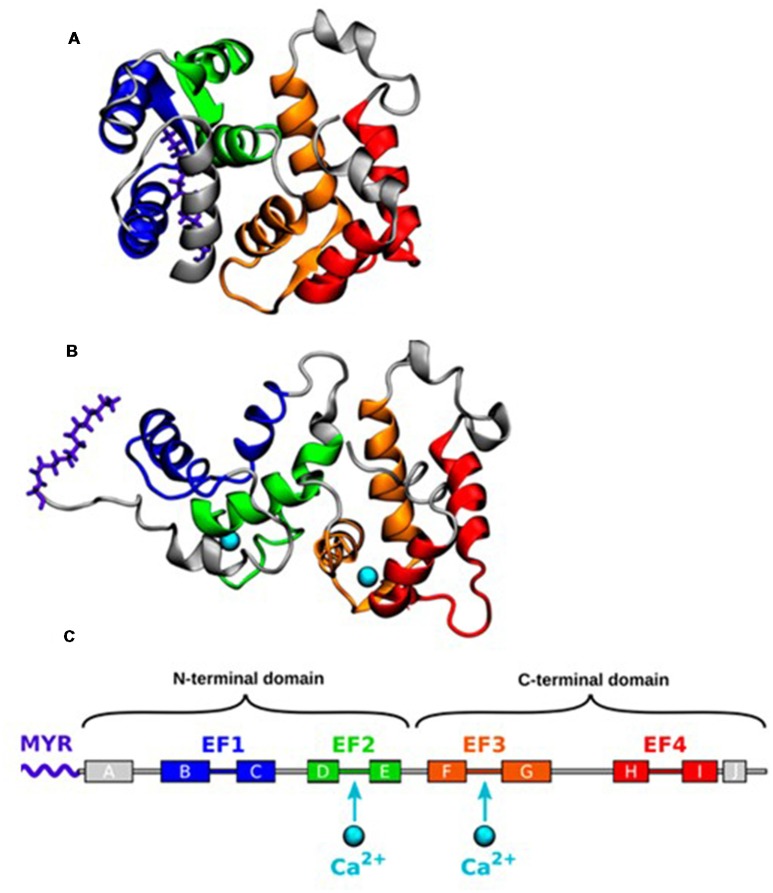
**(A)** Nuclear magnetic resonance (NMR) structure of the closed state of Recoverin (Rcv) with no calcium bound (geometry no. 1 of PDB ID 1IKU; Tanaka et al., 1995). **(B)** NMR structure of the open state with two calcium ions (Ca^2+^) bound (geometry no. 1 of PDB ID 1JSA; Ames et al., 1997). **(C)** The α-helices of Rcv are labeled alphabetically according to their order in the protein sequence. Adapted with permission from Timr et al. (2018). Copyright © 2018 American Chemical Society.

The three-dimensional structures of myristoylated Rcv in solution with no or 2 Ca^2+^ ions bound have been solved by nuclear magnetic resonance (NMR) spectroscopy (Tanaka et al., 1995; Ames et al., 1997). In the absence of Ca^2+^ binding, the myristoyl group is hidden in a deep hydrophobic pocket in the N-terminal domain, forming a compact structure termed as the T (tense) state (Figure 1). A protein conformational change is induced by the binding of two Ca^2+^ ions, which leads to the extrusion of the myristoyl group, forming an elongated structure termed as the R (relaxed) state. The Ca^2+^ induced exposure of the myristoyl group, known as Ca^2+^ myristoyl switch, allows Rcv binding to the phospholipid membranes only when intracellular Ca^2+^ concentration ([Ca^2+^]_i_) is high (Zozulya and Stryer, 1992; Dizhoor et al., 1993; Ames et al., 1995).

Recently, an intermediate conformational state, named as the I state, has been proposed following NMR relaxation dispersion and chemical shift analyses on ^15^N-labeled Rcv (Xu et al., 2011). The I state coexists with the structurally different T state ([I]/[T]>1%) on a millisecond time scale with its myristoyl group still buried inside the protein structure. NMR cannot demonstrate the exact structure because of its short lifetime, but molecular dynamics (MD) simulations describe a more detailed process underlining the importance of the I state (Timr et al., 2018). When [Ca^2+^]_i_ is low, Rcv fluctuates between the T and the I state. Stronger Ca^2+^ binding to the EF3 loop of I state stabilizes itself in response to the elevated [Ca^2+^]_i_ environment. Following the second Ca^2+^ binding to the EF2 loop, Rcv moves into the R state by performing the myristoyl switch.

Many different approaches have been applied to study the conformational change of Rcv in the past decade. Surface plasmon resonance (SPR) is known as a powerful tool to measure interaction modes of proteins, peptides, and lipids (Koch, 2012). The subtle conformational dynamics of Rcv induced by 100 nM to 600 nM of Ca^2+^ have been demonstrated by this method (Dell’Orco et al., 2010). This concentration is in the range of the estimated physiological [Ca^2+^]_i_ in intact photoreceptors, although some studies measure slightly lower values (Sampath et al., 1998, 1999; Woodruff et al., 2002). More importantly, SPR allows the simultaneous measurement of different Ca^2+^ binding proteins of the phototransduction cascade in response to the identical Ca^2+^ stimulus (Dell’Orco et al., 2012), mimicking the physiological condition during the light response. Double electron−electron resonance (DEER) analysis has also been used to demonstrate increased distance between two spin-labels at engineered cysteine residues on the Rcv surface upon Ca^2+^ binding, which indicates that Rcv forms dimer when [Ca^2+^]_i_ is high (Myers et al., 2013). Myers and colleagues proposed that Ca^2+^-induced dimerization of Rcv may interact with Rhodopsin which also forms a dimer in the membrane (Fotiadis et al., 2006; Knepp et al., 2012). Meanwhile Rcv dimer binds to two GRKs, which brings these molecules close to Rhodopsin dimer. When [Ca^2+^]_i_ drops upon light stimulation, Ca^2+^ free Rcv releases GRK, resulting in a rapid phosphorylation for Rhodopsin.

## Rcv-Disc Membrane Interaction

Many studies have demonstrated that the binding of Rcv to the membrane depends primarily on Ca^2+^ and the presence of the myristoyl group (Lange and Koch, 1997; Desmeules et al., 2002, 2007). When Ca^2+^ concentration is high, the extruded myristoyl group inserts into the disc membrane bilayer and allows the recruitment of Rcv at the membrane surface. However, the direct demonstration of membrane-bound Rcv is not available, because its three-dimensional structure is very hard to be determined by NMR in solution due to its slow reorientation rate. The membrane-bound Rcv structure as predicted by two-dimensional solid-state NMR suggests that the long molecular axis of Rcv is oriented at a 45° angle with respect to the membrane normal (Valentine et al., 2003). Several positively charged basic residues at the Rcv N-terminal are exposed closely towards the membrane surface. Although it is clear that the membrane insertion depends on both the presence of Ca^2+^ and the myristoyl switch, the detailed mechanism of how this is achieved is still elusive.

Numerous factors may regulate the Rcv binding process. Electrostatic interactions have become the focus of current investigation. Although neutral phosphatidylethanolamine and phosphatidylcholine constitute about 80% of the total phospholipid content in the rod outer segment disc membranes (Wu and Hubbell, 1993), Rcv preferentially binds to the negatively charged phosphatidylserine monolayers (Calvez et al., 2016). This preference disappears when the charges of the phosphatidylserine monolayer are shielded or the positively charged N-terminal residues of Rcv are mutated. This argues for a role of these residues in electrostatic interactions with the negatively charged phosphatidylserine in the presence of Ca^2+^. MD simulations support this notion, proposing that positively charged residues frequently contact the bilayer and that negatively charged phospholipids regulate the orientation of Rcv toward the membrane (Timr et al., 2017). On the other hand, deletion of 13 amino acids of the positively charged C-terminus does not change the binding property, although these residues have been proposed to be part of the electrostatic interactions (Senin et al., 2007).

The phospholipids in the rod outer segment disc membrane contain a high percentage of unsaturated acyl chains, suggesting that the disc membranes are highly fluid (Fliesler and Schroepfer, 1983). In order to assess the importance of membrane fluidity, Potvin-Fournier and colleagues compared membranes composed of the most abundant lipid phosphatidylcholine with either saturated or unsaturated acyl side chains (Potvin-Fournier et al., 2017). Solid-state NMR indicates that phosphatidylcholine with monounsaturated acyl chain (DOPC) produces the strongest Rcv immobilization, implying that Rcv binding favors the membrane with greater fluidity.

Another useful technique to study the Rcv-membrane interaction is site-specific fluorescent labeling (Yang et al., 2016). Traditional non-specific labeling for multiple lysines or specific single labeling for cysteine may damage Rcv structure and function. Especially Cys39, which is highly conserved in neuronal NCS proteins, has been shown to play an important role in regulating both Ca^2+^ and membrane binding of Rcv (Permyakov et al., 2012; Ranaghan et al., 2013). In this method, a p-azido-L-phenylalanine (AZF) group, which is a phenylalanine analog with an azide substituted in *para* position, is genetically engineered to replace either phenylalanine or tyrosine. In the case of Rcv, two phenylalanines are carefully selected because they have good accessibility to the solvent but do not affect membrane binding. Rcv is attached to a fluorescent probe 4-chloro-7-nitro-1,2,3-benzoxadiazole (NBD) *via* strain-promoted azide-alkyne cycloaddition. In this way, the membrane translocation of Rcv is visualized in a Ca^2+^ dependent manner and the membrane with negative spontaneous curvature or higher fluidity shows stronger Rcv binding in the presence of Ca^2+^.

Little is known about how the insertion of the myristoyl moiety affects the lipid membrane. Although the insertion does not seem to disturb membrane integrity (Potvin-Fournier et al., 2018), it greatly impacts the electrochemical properties of the bilayer (Brand and Koch, 2018). Both the surface charge density and the surface pressure of membranes are reduced upon Rcv binding, supporting the importance of electrostatic interactions.

## Ca^2+^-Induced Inhibition of GRK by Rcv

The extrusion of the myristoyl group induced by Ca^2+^ exposes a hydrophobic groove, which consists of residues highly conserved in all NCS proteins (Tanaka et al., 1995; Ames et al., 1997). NMR demonstrates that Rcv binds with a functional N-terminal fragment consisting of the first 25 amino acid residues of GRK (RK25) in the presence of Ca^2+^, indicating an interaction between the hydrophobic surface of RK25 amphipathic helix and Rcv hydrophobic groove (Ames et al., 2006). The same study also shows that Rcv-bound RK25, but not RK25 alone binds to immobilized Rhodopsin. Moreover, N-terminal deletion mutants of GRK still contain a functional catalytic domain but lose the ability to phosphorylate Rhodopsin (Higgins et al., 2006). These observations suggest that Ca^2+^-Rcv is positioned between GRK and Rhodopsin in a ternary complex to sterically obstruct Rhodopsin recognition without affecting GRK catalytic activity. On the other hand, the C-terminal region of Rcv (residues 190–202) has been implicated as an internal modulator of Ca^2+^ sensitivity and therefore may also affect GRK binding (Weiergräber et al., 2006). Indeed, Rcv mutants with various deletions of the C-terminal region show a lower affinity to the N-terminus of GRK and a weaker inhibition on GRK activity (Zernii et al., 2011). Moreover, mutating phenylalanine of the GRK N-terminus, which is predicted to contact the C-terminus of Rcv, leads to a strong reduction of binding to Rcv as demonstrated by SPR spectroscopy. These results indicate a direct involvement of the C-terminus in interaction between Rcv and GRK, providing another mechanism to regulate the inhibitory effect of Rcv.

Most studies investigated the biochemical properties of rod specific Rcv. There is very little information of Rcv variants present in cone photoreceptors. Such cone specific Rcv variants are found in all vertebrates with the exception of mammals (Yamagata et al., 1990; Kawamura et al., 1996; Arinobu et al., 2010; Zang et al., 2015).

Cone opsin in the zebrafish retina is phosphorylated in a Ca^2+^-dependent manner, suggesting a similar role of cone Rcv in the regulation of visual pigment phosphorylation during light response (Kennedy et al., 2004). In carp, cone Rcv binds to N-terminus of both rod and cone GRKs with similar affinity (Arinobu et al., 2010). Although the concentration of cone Rcv in the cone outer segment is estimated to be 20 times higher than that of rod Rcv in the rod outer segment, their inhibitory activity on both GRKs and their Ca^2+^ dependency *in vitro* is very similar. Judging on [Ca^2+^]_i_ measurements in darkness and determination of Rcv concentrations, the inhibition by cone Rcv is calculated to be 2.5 times higher than that by rods Rcv.

Interestingly, GRK is not the only binding partner of Rcv in the outer segment disc membrane. Caveolin-1, a major integral component of cholesterol-rich detergent-resistant lipid rafts of the rod disc membrane, has been shown to bind to Rcv in the absence of Ca^2+^ (Vladimirov et al., 2018). Although Caveolin-1 is not directly involved in phototransduction, its binding seems to increase Ca^2+^ affinity of Rcv, thereby allowing inhibition by Rcv even under low Ca^2+^ concentrations (Zernii et al., 2014). Therefore, Caveolin-1 binding may provide a mechanism which reserves small amounts of Rcv during bright light response or during light adaptation in outer segment membrane rafts and facilitates GRK inhibition upon Ca^2+^ change with high temporal resolution.

## Physiological Functions of Rcv in Phototransduction Cascade

**Figure 2:**
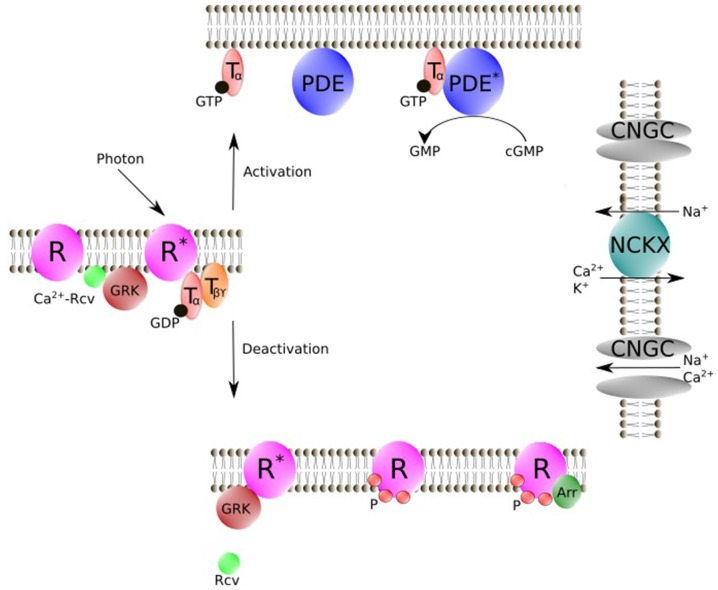
Schematic representation of vertebrate phototransduction cascade and the deactivation process for R* regulated by Rcv. Photon absorption activates R. R* then triggers the exchange of GTP for GDP on the T_α_. T_α_-GTP binds to cyclic nucleotide PDE. Stimulated PDE hydrolyzes free cyclic guanosine monophosphate (cGMP). In darkness, CNGC allows an influx of Na^+^ and Ca^2+^, while during illumination CNGC is shut off by cGMP decrease. NCKX is not affected by light, which results in a light-induced [Ca^2+^]_i_ decline. Rcv modulates phosphorylation of R* *via* GRK in a Ca^2+^ dependent manner. Phosphorylated R then is fully deactivated by binding of Arr. R, Visual pigment (inactive); R*, Light-activated visual pigment; T_α_, Transducin α subunit; T_βϒ_, Transducin β and ϒ subunits; PDE, Phosphodiesterase (inactive); PDE*, PDE-Transducin complex; NCKX, Na^+^/Ca^2+^, K^+^ exchanger, Arr, Arrestin; GRK, G protein-coupled receptor kinase; Rcv, Recoverin; CNGC, cyclic nucleotide–gated ion channels; P, Phosphorylation; Figure was drawn using Inkscape. Inkscape http://www.inkscape.org/.

The phototransduction cascade starts with the absorption of a photon by the visual pigment rhodopsin (Figure 2), which is a member of the G protein-coupled receptor family (Burns and Baylor, 2001; Fain et al., 2001; Lamb and Pugh, 2006; Fu and Yau, 2007). Activated rhodopsin (R*) interacts with the trimeric G protein transducin, which in turn binds to its target effector enzyme, phosphodiesterase (PDE), resulting in cyclic guanosine monophosphate (cGMP) hydrolysis. The decrease in cGMP concentration leads to the closure of cyclic nucleotide-gated ion (CNG) channels in the outer segment membrane, producing the electrical response to light. In darkness, CNG channels are partially open and there is a steady Ca^2+^ (and Na^+^) influx across the plasma membrane into the outer segment. This Ca^2+^ influx is reduced by the closure of the CNG channels during light response, while Ca^2+^ efflux *via* Na^+^/Ca^2+^ K^+^ exchanger continues, leading to a decrease in [Ca^2+^]_i_ in the outer segment. This decline in [Ca^2+^]_i_ modulates the quenching of the phototransduction cascade, which requires the shutoff of both active intermediates (R* and PDE*) and the resynthesis of cGMP. Under steady background illumination, the [Ca^2+^]_i_ decline is proportional to the reduction in the circulating current and is crucial for lower photosensitivity and faster response kinetics during this process. What are the targets of Ca^2+^? The light-induced decrease in [Ca^2+^]_i_ is postulated to accelerate phosphorylation of R* *via* Rcv, speed up cGMP synthesis *via* guanylyl cyclase activating protein (GCAP), and increase cGMP affinity of the CNG channel *via* calmodulin (Koch and Stryer, 1988; Hsu and Molday, 1993).

Despite the fact that Rcv has been extensively shown to inhibit GRK in a Ca^2+^ dependent manner to regulate the phosphorylation of R* *in vitro* (Kawamura, 1993; Chen et al., 1995, 2012, 2015; Klenchin et al., 1995; Kennedy et al., 2004; Chen C.-K. et al., 2010; Sakurai et al., 2011b; Invergo et al., 2013), its physiological function in the phototransduction cascade has remained controversial. Electrophysiological recordings on mouse rods lacking Rcv showed accelerated photoresponse decay, which is consistent with an inhibition of GRK by Rcv at high [Ca^2+^]_i_ in darkness and a reduced lifetime of R* in the mutants during light response (e.g., Makino et al., 2004; Chen C.-K. et al., 2010). However, other authors proposed that both Rcv and GRK participate in the regulation of PDE activity (e.g., Chen et al., 2012, 2015). In addition, Rcv accumulates largely in the inner segment, cell body and synapse, indicating that Rcv may have greater importance in some processes other than phototransduction, such as signal transmission (Sampath et al., 2005; Zang et al., 2015). Moreover, the affinity of Ca^2+^ to Rcv is rather low with an EC_50_ of about 3,000 nM and the light induced Ca^2+^ decline in the outer segment, which is in the range of 200 nM to 600 nM, may not even affect Rcv activity (Chen et al., 1995; Sampath et al., 1999; Woodruff et al., 2002), while a more recent study demonstrated a much lower EC_50_ (400–550 nM; Arinobu et al., 2010). Furthermore, cone homologs of Rcv have been identified in many animals and have been shown to work in a similar way as rod Rcv (Yamagata et al., 1990; Gray-Keller et al., 1993; Kawamura et al., 1996; Arinobu et al., 2010; Zang et al., 2015; Lamb and Hunt, 2018). However, the physiological importance of Rcv may very well differ between photoreceptor types or even among species, considering the difference between rods and cones (e.g., Rcv concentration and [Ca^2+^]_i_ dynamics).

## Rcv in Rod Phototransduction

Rod photoreceptors operate at low light intensities and are able to be activated by the absorption of a single photon. It has been suggested that PDE* deactivation (around 250 ms) is slower than R* decay (around 50 ms) and dominates the overall photoresponse recovery in mouse rods (Krispel et al., 2006; Tsang et al., 2006; Doan et al., 2009; Chen C.-K. et al., 2010; Gross and Burns, 2010), but see (Doan et al., 2009). If the major role of Rcv is to inhibit GRK, preventing the phosphorylation of R* when Ca^2+^ is high, its deletion should result in a shortened lifetime of R* and decrease the gain of signaling pathway. This in turn should lead to lower light sensitivity, without strongly affecting the time course of response decay. However, electrophysiological recordings from Rcv^−/−^ mice showed an accelerated dark-adapted flash response, but only a minor effect on response sensitivity (Makino et al., 2004; Sampath et al., 2005; Chen J. et al., 2010). Although phosphorylation is essential for R* shutoff (Chen et al., 1999), the phosphorylation regulated by Rcv seems to have a limited impact on photosensitivity, which corresponds to little effect of GRK expression levels on dim light amplitude in mice. Interestingly, absence of Rcv reduces around 50% of single photon response amplitude in GCAPs^−/−^ background, which indicates that the sensitivity regulation mediated by Rcv may be masked by a more powerful feedback mediated by GCAPs (Makino et al., 2004; Vinberg et al., 2015). However, both the dominant time constant (τ_D_) and the exponential decay constant of the response (τ_REC_) are significantly accelerated in Rcv^−/−^ rods (Chen et al., 2012, 2015). In order to reduce τ_D_, the effect of Rcv depletion must be mediated *via* shortening the lifetime of the rate-limiting step. PDE* decay has been proven to still remain rate-limiting for response recovery both in Rcv^−/−^ rods in WT background and in Rcv^−/−^ rods with delayed PDE* quenching. In this case, a novel role of Rcv regulating PDE* deactivation is the most parsimonious explanation.

During light adaptation, rod sensitivity is reduced and response recovery is accelerated when background illumination increases, which extends the working range of rods to brighter light levels (Fain et al., 2001). Rcv, GCAP and Calmodulin are all Ca^2+^ binding proteins, which have been proposed to contribute to the light adaptation. Surprisingly, deletion of Rcv or Calmodulin binding site on CNG channels has little impact on sensitivity and removal of GCAPs affects only some but not all the changes in sensitivity during background adaption. Furthermore, no impact of Calmodulin binding site deletion in GCAPs^−/−^ rods is observed (Chen J. et al., 2010). Perfusing rods with low Ca^2+^ solution in darkness can mimic the [Ca^2+^]_i_ reduction during light adaptation (Vinberg et al., 2015). This method can decrease the single photon response amplitude of GCAPs^−/−^ rods and GCAPs^−/−^ Rcv^−/−^ rods to the same level, about 25% of the value in GCAPs^−/−^ rods in normal solution. This may suggest other Ca^2+^ dependent mechanism(s).

In the case of time course, an increase in the background intensity produces a speed up in light response recovery and a progressive decrease in τ_D_ nearly in proportion to background intensities (Fain et al., 2001; Woodruff et al., 2008; Chen et al., 2015). Deleting PDE_ϒ_ subunit eliminates this gradual reduction in τ_D_, suggesting that the accelerated response recovery during light adaptation primarily depends on PDE* turn off. Removal of Rcv from the genome yields the same effect. Moreover, τ_D_ produced by different background intensities in Rcv^−/−^ rods is very close but slightly longer than Rcv^+/+^ rods under the brightest ambient intensity, indicating the regulation of Rcv on PDE* contributes the majority of the acceleration during light adaption, but some other mechanism(s) may still be involved. Is this mechanism Ca^2+^ dependent? Background light can still accelerate the response in Rcv^−/−^ rods recorded in low extracellular Ca^2+^ concentration ([Ca^2+^]_o_) which in theory can clamp [Ca^2+^]_i_ to its maximal light-adapted level, indicating a possible Ca^2+^-independent process (Vinberg et al., 2015). Furthermore, during background light exposure, photoresponse integration time declines while the circulating current gradually increases (Morshedian et al., 2018). Both effects work to increase sensitivity at dim light condition near the threshold and improve the temporal resolution under bright light condition, but these effects disappear in Rcv^−/−^ rods. An effect on spontaneous and light activated PDE lifetime may contribute to these observations.

In short, Rcv can only regulate photosensitivity in the absence of Ca^2+^ feedback mediated *via* GCAPs in mouse rods. Another Ca^2+^ sensitive mechanism, other than mediated by Calmodulin, must be involved in this regulation. Rcv modulation on PDE* contributes the majority of the regulation on response kinetics.

How exactly Rcv may work on PDE* is still unknown. Most likely its regulation works still *via* GRK, because the change in photoresponse kinetics is very similar in rods overexpressing GRK and in rods without Rcv (Sakurai et al., 2011b; Chen et al., 2012, 2015). Most likely Rcv binds to Ca^2+^ and inhibits GRK in darkness, while during light response, Ca^2+^-free Rcv releases from GRK, enabling phosphorylation of target protein(s) and the deactivation of PDE*. GTPase-activating proteins (GAPs) accelerate the turn off rate of Transducin. Deletion of GRK in GAPs deficient rods produces little effect on the time course of response, suggesting that the target protein(s) may be involved in the PDE-Transducin pathway (Chen et al., 2015). However, there is no biochemical evidence to support this novel function of GRK and the electrophysiological recordings from rods overexpressing GRK show contradictory results in different studies. For example, a threefold overexpression of GRK is able to speed up the response kinetics of dim light (indicated by τ_REC_) but not saturating light (indicated by τ_D_; Sakurai et al., 2011b). Because τ_D_ is unaffected, GRK most likely regulates R* decay which is certainly shorter than the lifetime of the rate-liming step PDE*, but close enough to affect the overall dim light response recovery. In contrast, when GRK is 12-fold overexpressed, both τ_REC_ and τ_D_ are reduced, suggesting a direct modulation of GRK on PDE* (Chen et al., 2012). Furthermore, a mathematical model of phototransduction partially predicts the experimental effects of GRK downregulation and overexpression on response kinetics without consideration of an interaction between GRK and PDE* (Invergo et al., 2013). Therefore, the possible mechanism underlining GRK regulation needs to be further investigated.

Rcv has indeed been shown to regulate the lifetime of R* in intact rods. When GRK1 is overexpressed and R* turnoff becomes rate-limiting for light response termination (Chen C.-K. et al., 2010), response acceleration during light adaptation disappears in Rcv^−/−^ rods. However, together with other studies mentioned above, the regulation of Rcv on PDE* may play a more significant role.

Interestingly, the situation in amphibian rods appears somewhat different. The overall response recovery or the rate-limiting step is insensitive to [Ca^2+^]_i_ (Lyubarsky et al., 1996; Matthews, 1996). However, there is a Ca^2+^ sensitive step early in the light response, which can be prolonged to dominate photoresponse kinetics by prolonging the lifetime of R* (Matthews et al., 2001). Therefore, unless there is some novel mechanism, this Ca^2+^ sensitive step most likely represents the Rcv mediating phosphorylation of R*, while the lifetime of PDE* which seems to dominate the response recovery in amphibian rods may not regulated by Ca^2+^ (Nikonov et al., 1998). This proposal is also supported by the fact that τ_D_ is insensitive to different background lights in salamander rods (Pepperberg et al., 1992; Nikonov et al., 2000), in stark contrast to the Ca^2+^ sensitive τ_D_ and background sensitive τ_D_ in mice (Woodruff et al., 2008; Chen et al., 2015; Vinberg et al., 2015).

## Rcv in Cone Phototransduction

Cone photoreceptors are mainly responsible for daytime vision and mediate color vision (Fu and Yau, 2007). They share a similar G-protein signaling pathway with rods, but their response is characterized by lower sensitivity and faster kinetics. Cones are capable to operate over a nine-order of magnitude intensity range, which is much larger than the range typically observed in rods. In addition, cones do not saturate under bright background illumination, suggesting a more powerful light adaptation mechanism (Baylor and Hodgkin, 1974; Matthews et al., 1990; Schneeweis and Schnapf, 1999). Indeed, light induced Ca^2+^ decline is much faster and the Ca^2+^ dynamic range is three times wider in cones than in rods (Sampath et al., 1999, 1998; Woodruff et al., 2002). Ca^2+^ regulation on GC *via* GCAPs is much weaker in mouse cones than rods (Sakurai et al., 2011a). Furthermore, the elevated cGMP turnover observed in background light, which accounts for many adaptive changes in salamander rod response, is estimated to be much higher in rods than in cones (Cornwall and Fain, 1994; Cornwall et al., 1995; Hodgkin and Nunn, 1988; Nikonov et al., 2000). Therefore, the Ca^2+^ feedback on GCAPs seems not to contribute to the difference between photoreceptor types during light adaptation. In contrast, the range of Ca^2+^-dependent regulation of cone homolog of GRK (GRK7) activity in cones is more than 100 times greater than the range of regulation of rod homolog of GRK (GRK1) activity in rods, suggesting that the phosphorylation mediated by Rcv may contribute more during light adaption in cones (Arinobu et al., 2010). Overall, the details of Rcv modulation in cone photoreceptors are not fully understood.

Mammalian photoreceptors share the same Rcv. Mouse photoreceptors even share the same GRK (GRK1), therefore, they presumably work together in a similar way in both cell types. Electroretinography (ERG) on Rcv deficient cones shows not only an accelerated response recovery, but also an around twofold decrease in photosensitivity in darkness, which differs from rods (Sakurai et al., 2015). This result is consistent with *in* *vitro* evidence for light-dependent phosphorylation of cone opsin by GRK1 (Zhu et al., 2003). During light adaptation, photosensitivity is clearly lower in Rcv^−/−^ cones than in controls in dim background light and becomes identical under bright light conditions (Sakurai et al., 2015). On the one hand, it is likely that the bright background light reduces [Ca^2+^]_i_ to a certain level that all the Rcv becomes Ca^2+^ free in control and has no ability to inhibit GRK. On the other hand, it is also possible that the spontaneous decay primarily contributes to quench the cone opsin when the light intensity is very high. This possibility is consistent with the independency of response kinetics on the expression levels of GRK when high percentage of cone pigment is bleached, which is demonstrated in the same study. Nevertheless, the response sensitivity continues to decrease in both mutant and control when background intensity increases further. These observations suggest that additional mechanism(s) must be at work to regulate the photoresponse, for example *via* GCAPs (Sakurai et al., 2011a). Surprisingly, the effect of Rcv removal is similar to GRK1 knockdown but opposite to GRK1 overexpression in cones. GRK1 knockout slows down the photoresponse as expected. The turning point between GRK1 knockdown and knockout is unknown, making it hard to estimate the molecular mechanism underlining this unexpected response kinetics. In other mammalian cones, the situation may be even more complicated with an additional cone homolog of GRK (GRK7) being present.

In salamanders, single cell recording indicates that cone photoresponse decay is dominated by the quenching of cone opsin and it is sensitive to [Ca^2+^]_i_ (Matthews and Sampath, 2010; Zang and Matthews, 2012). Although this Ca^2+^ sensitive process is not specified, cone opsin phosphorylation mediated by Rcv seems probable and this process allows Ca^2+^ directly controlling the lifetime of rate-limiting step, in contrast to salamander rods.

In zebrafish, cone opsin phosphorylation shows strong light and Ca^2+^ dependence (Kennedy et al., 2004). Four Rcvs have been identified in zebrafish (Zang et al., 2015). All four of them are expressed in cones with only one of them existing in rods as well. ERG response recovery accelerates in Rcv(s) knockdown animals and some Rcvs operate at varying light intensities, suggesting different Ca^2+^ sensitivity and (or) GRK affinity among Rcvs. Indeed, a recent biochemical work shows different Ca^2+^ affinity and Ca^2+^-induced conformational changes among zebrafish Rcv isoforms and bovine Rcv (Elbers et al., 2018). Interestingly, the amino acids at the critical positions of Ca^2+^ binding sites are completely conserved across species with the exception of one zebrafish Rcv, where only three out of four positions are conserved in EF2 (Lamb and Hunt, 2018). This may at least partially contribute to their different molecular properties. Downregulation of GRK7 largely delays the response and Rcv knockdown in this background has no effect, consistent with the function of Rcv to inhibit GRK *in vitro* (Rinner et al., 2005). Notably, the effect of GRK7 knockdown in zebrafish seems to work the opposite way when compared to the situation in mouse cones, but the downregulation level here is around 95% and may represent the situation of GRK1^−/−^ in mice. Therefore, whether GRKs function the same way in mouse and zebrafish cones is still unknown.

In summary, Rcv certainly plays a role in regulating the cone phototransduction decay. However, as the popular animal models of mouse and bovine have rod-dominant retina, our knowledge about the function of Rcv in cones is very limited. Different roles of Rcv in rods and cones may very well underlie overall differences in their photoresponse.

## Role of Rcv in Signal Transmission

Rcv is expressed in all photoreceptors and also in some subtypes of bipolar cells (Dizhoor et al., 1991; Milam et al., 1993; Haverkamp and Wässle, 2000; Strissel et al., 2005; Zang et al., 2015). Most of Rcv has been quantitatively shown to accumulate in rod inner segment in both light and dark adapted retina in mice (Strissel et al., 2005). Light induces a remarkable translocation of Rcv from the outer and inner segments towards the synaptic terminals. Interestingly, GRK remains in the outer segments independent of illumination. Those observations are not only consistent with the well-documented role of Rcv releasing GRK upon [Ca^2+^]_i_ reduction in the outer segment, but also indicate some other function(s) of Rcv which is independent from GRK in other cellular compartments of photoreceptors.

Indeed, although the light sensitivity is not affected in Rcv^−/−^ rods, the sensitivity of rod-mediated vision is reduced in the behavior assay (Sampath et al., 2005). The same study also demonstrate that the dim light-evoked response of rod bipolar cells and ganglion cells, which do not express Rcv, is shortened in Rcv knockout mice. This decreased response time is produced by the reduced signal transfer from rods to rod bipolar cells instead of rod phototransduction itself.

## Conclusion

Here, we reviewed the molecular process underlining Ca^2+^-induced membrane and GRK binding of Rcv. Moreover we summarized the physiological function of Rcv in regulating phototransduction cascade and in signal transmission. Rcv is clearly capable of regulating phototransduction decay in dark-adapted as well as light-adapted response, but the molecular mechanisms are still not entirely clear. Biochemical evidence supporting or excluding the modulation of Rcv on PDE* is required. Moreover, animal models favored by both biochemists and electrophysiologists have traditionally been rod-dominant. Hence our knowledge of cone phototransduction regulation lags behind. Therefore, the importance of Rcv cone homolog(s) and the potential function difference between rod and cone Rcv will need to become a focus of future research.

## Author Contributions

JZ and SN drafted and revised the review article.

## Conflict of Interest Statement

The authors declare that the research was conducted in the absence of any commercial or financial relationships that could be construed as a potential conflict of interest.

## Abbreviations

Ca^2+^: calcium ions
Rcv: Recoverin
GRK: G protein-coupled receptor kinase
[Ca^2+^]_o_: extracellular Ca^2+^ concentration
[Ca^2+^]_i_: intracellular Ca^2+^ concentration
GC: guanylyl cyclase
GCAP: guanylyl cyclase activating protein
R*: light-activated visual pigment
PDE*: PDE-Transducin complex
GAPs: GTPase-activating proteins
cGMP: cyclic guanosine monophosphate
CNG channels: cyclic nucleotide-gated ion channels
NMR: nuclear magnetic resonance spectroscopy
SPR: surface plasmon resonance
DEER: double electron−electron resonance
NBD: 4-chloro-7-nitro-1,2,3-benzoxadiazole.
